# Hallucinogenic potential: a review of psychoplastogens for the treatment of opioid use disorder

**DOI:** 10.3389/fphar.2023.1221719

**Published:** 2023-08-22

**Authors:** Mary G. Hornick, Ashley Stefanski

**Affiliations:** College of Science, Health and Pharmacy, Roosevelt University, Schaumburg, IL, United States

**Keywords:** opioid use disorder, psychoplastogens, ibogaine, ketamine, psychedelic agents

## Abstract

The United States is entering its fourth decade of the opioid epidemic with no clear end in sight. At the center of the epidemic is an increase in opioid use disorder (OUD), a complex condition encompassing physical addiction, psychological comorbidities, and socioeconomic and legal travails associated with the misuse and abuse of opioids. Existing behavioral and medication-assisted therapies show limited efficacy as they are hampered by lack of access, strict regimens, and failure to fully address the non-pharmacological aspects of the disease. A growing body of research has indicated the potential of hallucinogens to efficaciously and expeditiously treat addictions, including OUD, by a novel combination of pharmacology, neuroplasticity, and psychological mechanisms. Nonetheless, research into these compounds has been hindered due to legal, social, and safety concerns. This review will examine the preclinical and clinical evidence that psychoplastogens, such as ibogaine, ketamine, and classic psychedelics, may offer a unique, holistic alternative for the treatment of OUD while acknowledging that further research is needed to establish long-term efficacy along with proper safety and ethical guidelines.

## Introduction

According to the 2021 National Survey on Drug Use and Health, an estimated 9.8 million individuals misused pain relievers and/or heroin and approximately 5.6 million individuals suffered from opioid use disorder (OUD) in the previous year alone (SAMHSA, 2022). Since the 1990s, opioid use in the United States has skyrocketed from an initial wave caused by an increase in opioid prescriptions to a rapid surge in heroin use a decade later, and a steady influx of fentanyl and other synthetic opioids over the past 10 years. Overdose deaths involving opioids have significantly increased along with their use and abuse, rising from 50,000 to over 80,000 annually from 2019 to 2021, respectively (NIDA, 2023). Along with preventable deaths, the misuse and abuse of opioids is associated with comorbid mental health disorders beyond addiction, legal and financial difficulties, and significant disruptions in familial and social relationships. Indeed, of the 7.7 million Americans over the age of 18 suffering from severe mental illnesses, 6.4 million people had a comorbid substance use disorder (SUD), with 10.3% of those involving the misuse of opioids (SAMHSA, 2022). These numbers make it abundantly clear that any treatment of OUD must also address the possible concomitant mental illness(es) affecting the patient in order to be successful.

Current treatments for OUD mainly focus on alleviating withdrawal and craving symptoms through the use of opioid agonists. Despite these opioid agonist therapies (OATs) being considered the gold standard treatment for OUD, there is an extraordinarily high rate of relapse (Weiss et al., 2011; Amato et al., 2013; Bentzley et al., 2015; Hser et al., 2016). Indeed, a review of buprenorphine maintenance therapy studies found that relapse to illicit opioid use was greater than 50% in every study reviewed, while those with a comparable tapering duration of 4 weeks varied widely, demonstrating the maintenance of abstinence rates from 9.6% to 50% (Bentzley et al., 2015). Similarly, a comparison of buprenorphine/naloxone *vs.* methadone indicated that 50.9% and 41.1% of patients, respectively, had used heroin or opiates during the 60-month follow-up (Hser et al., 2016). Extending the maintenance and tapering phases of treatment has been shown to increase initial success rates, but the addition of opioid drug counseling failed to significantly improve outcomes either immediately following the treatment (46.7% *vs.* 51.7%) or after an 8-week follow-up (7.2% *vs.* 10%) (Weiss et al., 2011). Although there are a number of reasons for the failure rate of these treatments, the most common themes seem to be the required long-term, strict adherence to the regimen and the inability of the pharmacological intervention, even with adjunct counseling, to address other aspects of OUD beyond addiction. Additionally, opioid agonist therapy itself is not without risk, as indicated by the ∼4% of monthly overdose deaths that involve methadone itself (Jones C. M. et al., 2022). A new approach is therefore needed to treat and meet the needs of those suffering from OUD, for whom OATs have either failed or are incompatible.

The following question is then raised: what would an idealized treatment for OUD look like? Beyond the standards of safety and efficacy, an ideal treatment would reduce or eliminate withdrawals and cravings; be more accessible via time, effort, and financial considerations; and provide patients with assistance in the more ineffable and variable aspects of the disease, such as comorbid mental health conditions and addiction-related relationship difficulties. Although there is not likely to be a magic-bullet, one-size-fits-all treatment that meets all of these requirements, there is a class of drugs that, along with psychotherapy, does offer hope to those suffering with OUD.

Drugs that rapidly promote induced neuroplasticity, termed “psychoplastogens,” may hold the key to the future of OUD treatment. Although a number of drugs are capable of inducing neuro-, dendrito-, and spinogenesis, the term “psychoplastogen” was coined by David Olson’s lab in 2018 from the Greek roots for mind (psyche), molding (plast), and producing (gen) to highlight the ability of certain drugs to promote both structural and functional neuroplasticity in a (presumably) therapeutic manner. Compounds in this category include hallucinogens, such as ibogaine, ketamine, and classic psychedelics, along with non-hallucinogenic analogs of these molecules (Ly et al., 2018). Although each of these drugs has its own unique pharmacology, a number of recent preclinical reports highlight their similarity in the ability to increase structural and functional connectivity, specifically within the prefrontal cortex (PFC) (Ly et al., 2018; Olson, 2022; van Elk and Yaden, 2022; Vargas et al., 2023). Atrophy and dysfunction of neurons within the prefrontal cortex are hallmarks of a variety of neuropsychiatric disorders, particularly those affected by stress, such as depression and addiction (Goldstein and Volkow, 2011; Vargas-Perez et al., 2014; Hare and Duman, 2020; Duman et al., 2021). Although a smattering of anecdotal and clinical evidence for the rapid efficacy of these compounds in treating depression, anxiety, and substance use disorders arose as early as the 1950s, their recreational use and the resulting legal crackdown have stifled research in this area until recently. Indeed, it remains to be seen if psychoplastogen-induced neuroplasticity (or part of it) is the driving force behind the apparent clinical efficacy of these compounds.

Within the past 25 years, there has been a resurgence of interest in these compounds for the treatment of a number of neuropsychiatric diseases, with preliminary clinical trials indicating the promise they hold for depression (Ahmed et al., 2023; Goodwin et al., 2023), post-traumatic stress disorder (Mitchell et al., 2021; Abdallah et al., 2022), and addiction (Brown and Alper, 2018; Noller et al., 2018; Garcia-Romeu et al., 2019; O'Shaughnessy et al., 2021; Bogenschutz et al., 2022), among others. The appeal and potential efficacy of psychoplastogens for these disorders is related to their rapid, long-lasting effects on not only the symptoms associated with the primary disorder but also to the alterations in patients’ attitude and approach to daily life and relationships, following as little as a single dosing session. In this review, therefore, we examine the preclinical and clinical evidence of the potential of psychoplastogens, such as ibogaine, ketamine, and classic psychedelics (lysergic acid diethylamide (LSD), psilocybin, and ayahuasca), in addressing not only the primary addiction but also the multi-faceted complexities associated with OUD in order to treat the patient as a whole.

## Ibogaine

Perhaps the most well-studied psychoplastogen for the treatment of OUD, specifically, ibogaine is an indole alkaloid found in the root bark of the African shrub *Tabernanthe iboga*. Utilized for centuries by those who follow the Bwiti religion of Central and Western Africa; shavings of the iboga root bark are taken in small amounts to stave off fatigue, thirst, or hunger, while larger amounts are used to induce a trance-like state during religious and initiation ceremonies (Brown, 2013). The iboga root was brought to Europe in the early 20th century, where ibogaine was isolated, purified, and eventually sold in France as the psychostimulant “Lambarene” to treat fatigue and depression.

The potential of ibogaine for combating opioid dependence was discovered (from a non-indigenous perspective) not in the clinic but, like so many hallucinogenic drugs in the 1960s, through recreational experimentation. Hoping to experience a psychoactive trip, Howard Lotsof and 19 other individuals, seven of whom were dependent on heroin, ingested hallucinogenic doses of ibogaine (Alper et al., 2001). Noting that withdrawals and cravings for heroin did not occur during or immediately after the experience, five of the heroin-dependent seven people remained abstinent from opioids for at least 6 months. Nevertheless, despite the advocacy of Lotsof, and scientists and psychiatrists around the country, who were utilizing ibogaine for a variety of psychiatric conditions, the possession of ibogaine became illegal in 1967, followed shortly after by its designation as a Schedule I drug with the passage of the Controlled Substances Act in 1970 (Alper et al., 2001).

However, research, advocacy, and even unregulated treatments, involving the use of ibogaine to treat addiction, quietly continued in various parts of the world. To date, it is estimated that over 10,000 patients have been treated with ibogaine worldwide (Luz and Mash, 2021). A number of published reports, including some clinical trials, have shown that Lotsof was correct—ibogaine does appear to reduce withdrawal and craving while allowing a significant number of patients to remain abstinent from opioids for an extended period of time after a single treatment (Table 1).

**TABLE 1:** Selected published reports of ibogaine administration in patients with OUD. SOWS, Subjective Opioid Withdrawal Scale; ASIC, Addiction Severity Index composite; BDI, Beck Depression Inventory; COWS, Clinical Opioid Withdrawal Scale; BSCS, Brief Substance Craving Scale.

Study type	N	Ibogaine dosage	Post-treatment assessment period	Outcome
Clinical trial (Alper et al., 1999)	33	6–29 mg/kg	≤72 h	75% resolution of withdrawal without drug seeking behavior
Clinical trial (Mash et al., 2000)	27	8–12 mg/kg	≤14 days; 1 month	Decreased cravings and self-reported decrease in depressive symptoms
Case report (Cloutier-Gill et al., 2016)	1	2.5–20 mg/kg (32 mg/kg total)	18 months	Sustained abstinence attributed to a “spiritual awakening”
Observational study (Brown and Alper, 2018)	30	3–12 mg/kg (avg total 1,540 mg)	1, 3, 6, 9, and 12 months	Improved SOWS (*p* <0.001)
				Significant reduction in the ASIC for drug use, family/social status, and legal status at all timepoints
				Sustained abstinence at 1 (50%), 3 (33%), 6 (20%), 9 (37%), and 12 (23%) months
Observational study (Noller et al., 2018)	14	25–55 mg/kg	12 months	Improved SOWS (*p* = 0.015)
				Significant reduction in the ASIC-lite for drug use and family/social status
				Reduction in BDI-II (*p* <0.001)
				Sustained abstinence at 3 (87.5%), 6 (85.7%), and 12 (75%) months
Retrospective study (Davis et al., 2018)	73	15 ± 5 mg/kg	≥1 year	Sustained abstinence, 36%; decreased opioid use, 45%
				Treatment responders reported persisting positive personal and social effects along with heightened spiritual awareness *vs.* non-responders
Retrospective study (Malcolm et al., 2018)	50	18–20 mg/kg	≤48 h	Improved SOWS, COWS, and BSCS with a 78% resolution of withdrawal and 79% minimal craving
Case series (Wilson, 2020)	2	25.5 mg/kg; 13–34 mg/kg	3 years; 2 years	Sustained abstinence for 3 and 2 years, respectively

### Potential mechanisms of action for ibogaine as a treatment for OUD

So how is ibogaine able to affect such a rapid, drastic change in OUD patients, many of whom had previously tried and failed to remain abstinent using the gold-standard opioid agonist treatment? Researchers are still trying to answer that question, with most data supporting a complex blend of pharmacological action, neuroplasticity, and oneirophrenic or holotropic experiences.

Ibogaine exhibits complex pharmacological action at a wide range of neurotransmitter receptors, with a low binding affinity for the serotonin reuptake transporter; sodium channels; nicotinic acetylcholine; N-methyl-D-aspartate (NMDA) glutamate; and kappa-opioid, mu-opioid, and sigma-2 receptors (Brown, 2013). Although it is an agonist for 5-HT_2A_, 5-HT3, and muscarinic receptors, ibogaine is also an antagonist for NMDA glutamate and nicotinic acetylcholine receptors. Ibogaine, which is highly lipophilic, therefore, concentrates in the brain and adipose tissue and slowly metabolizes via demethylation to form the active metabolite, noribogaine, which has its own unique pharmacological profile. Noribogaine, with a circulating half-life of 28–49 h, exhibits less affinity for NMDA receptors and a greater affinity for mu-opioid receptors than its parent compound (Mash et al., 2000). This agonism of opioid receptors by noribogaine may contribute to the observed reductions in withdrawals and cravings.

Although noribogaine’s actions at opioid receptors may mediate initial therapeutic effects relating to craving and withdrawal symptoms, they do not fully explain the long-lasting anti-addiction effects often noted after a single session of ibogaine treatment for opioid dependence. It has been postulated that the enduring anti-addictive properties of ibogaine relate instead to a rewiring of neural circuitry associated with addiction. Indeed, both ibogaine and noribogaine increase the glial-derived neurotrophic factor (GDNF) in the ventral tegmental area, which has been shown in preclinical studies to reduce self-administration of alcohol (He et al., 2005). Interestingly, however, 18-methoxycoronardine (18-MC), a structural analog developed to mitigate the negative cardiovascular and hallucinogenic side effects of ibogaine, does not increase the GDNF despite demonstrating efficacy in reducing cocaine and morphine self-administration (Brown, 2013). Noribogaine, but not ibogaine, on the other hand, has been shown to increase dendritic arbor complexity and promote neuritogenesis in cortical neurons *in vivo*. This rapidly induced neuroplasticity is mediated via the stimulation of TrkB, mTOR, and intracellular 5-HT_2A_ signaling pathways and is thought to be essential to the therapeutic effects of ibogaine and other psychoplastogens on complex mental health conditions, such as depression and SUD (Ly et al., 2018; Vargas et al., 2023).

Similar to classic psychedelics, the activation of the 5-HT_2A_ receptors is also thought to be responsible for the hallucinatory effects of ibogaine. The ibogaine experience has been described both as oneirophrenic, or a waking dream-like state, and holotropic, a healing transformation of the consciousness (Underwood et al., 2021). Although Bwiti practitioners describe the ibogaine visionary experience in four phases, current researchers typically identify three distinct stages. The four phases of the Bwiti ibogaine initiation ritual are broken down by the type of vision and its attached significance: 1) random images lacking meaning; 2) fractal animal or natural images; 3) mythical images promoting peace; and 4) encounters with spiritual entities or ancestors. The three stages identified by researchers in conjunction with the treatment for SUD, in contrast, are characterized by both physical and psychological effects: 1) an acute phase defined by ataxia, possible GI distress, and changes in the heart rate while revisiting memories; 2) a reflective phase focusing inward while examining alternative pasts and futures; and 3) a residual stimulation phase with a decreased intensity of visions and a gradual return to the external environment (Alper et al., 2001; Underwood et al., 2021). It is possible that this altered state of consciousness is a necessary component to ibogaine’s anti-addictive therapeutic effects. Although the vast majority of ibogaine patients claim the experience to be both psychologically difficult and physically draining, objective and subjective reports from these same patients describe how it increased empathy, self-reflection, and appreciation, and decreased their feelings of anxiety and depression, improving familial and social relations (Brown and Alper, 2018; Camlin et al., 2018; Davis et al., 2018; Malcolm et al., 2018; Noller et al., 2018; Brown et al., 2019; Wilson et al., 2020). As one patient reflected, “I was also able to begin to see how, because I felt so unlovable, I paid in one way or another for love. I was able to stop that and open myself up to real love for maybe the first time in my adult life” (Brown et al., 2019). Consideration of these reports is increasingly important as researchers develop new compounds based off of hallucinogens, such as 18-MC and tabernanthalog (TBG). Although these novel molecules are showing some promise in preclinical studies regarding neuroplasticity and reduced addiction-seeking behaviors (Brown, 2013; Cameron et al., 2021), the lack of an altered state of consciousness could render them less effective or even completely ineffective when administered to human patients. This is an active area of research that requires further study.

### Safety, guidelines, and future considerations for ibogaine in the treatment of OUD

Despite the large numbers of patients seeking ibogaine treatment for SUDs like OUD, its status as a Schedule I drug in the United States has hindered the ability of researchers to properly study its efficacy and safety and to create guidelines for its use. Ibogaine, while non-addictive and generally considered safe in the doses utilized for SUD treatment, does have known adverse and potentially dangerous side effects. It is known to inhibit cardiac hERG potassium channels, leading to QT interval prolongation and an increased risk for potentially fatal arrhythmias, including torsades de pointes (TdP) (Koenig and Hilber, 2015; Luz and Mash, 2021). The IC_50_ concentration for hERG channels is approximately four times greater than the estimated therapeutic levels of ibogaine. However, one must consider that ibogaine is metabolized to noribogaine primarily by cytochrome P450 2D6 (CYP2D6) enzymes, which not only plays an important role in terms of drug–drug interactions but also in terms of genetic variations. For instance, approximately 10% of the Caucasian population lacks CYP2D6, which could significantly put them more at risk for adverse cardiovascular effects due to unexpectedly high plasma levels of ibogaine (Koenig and Hilber, 2015).

Since the beginning of the “vast uncontrolled experiment” of patients flocking to (mostly) unregulated clinics for ibogaine treatment, at least 32 ibogaine-related deaths have been reported (Luz and Mash, 2021). Although most of the reported fatalities were attributed to the aforementioned adverse cardiovascular effects, it is important to note that they also occurred in unsafe and unregulated settings with improper medical screening and dosing along with a lack of suitable medical care on site for monitoring and emergencies. In response to these severe and potentially dangerous side effects, guidelines for the use of ibogaine to treat SUDs have been published (Dickinson et al., 2016). These days, despite the continued lack of federal regulation, most providers understand the importance of careful patient screening for medical (e.g., EKG and liver function) and psychiatric exclusion criteria prior to treatment, as well as the proper monitoring and availability of medical assistance during and after treatment. Utilizing this careful approach, a recent study by Knuijver et al. was able to demonstrate that, while ibogaine treatment for OUD may produce clinically relevant bradycardia, QTc prolongation, and severe ataxia, these side effects were all manageable and reversible (Knuijver et al., 2022).

Further research is necessary before ibogaine treatment for SUDs in general or OUD, specifically, can be approved and made available to the American public, but it is undeniable that this unique, holotropic psychoplastogen holds promise for those suffering from these conditions. As previously mentioned, researchers are attempting to synthesize ibogaine analogs (18-MC and TBG) with improved safety profiles, but, to date, there has only been a single human trial with either of these compounds (NIH, 2023). There are currently two clinical trials in the recruiting stages for ibogaine for the treatment of opioid withdrawal and detoxification (Table 2). It will be extremely interesting to see if the non-hallucinogenic analogs produce the same efficacy as their hallucinogenic counterpart and if a regulated study design can mitigate any adverse effects of ibogaine itself.

**TABLE 2:** Current clinical trials of psychoplastogens for the treatment of OUD (NIH, 2023).

Drug	NCT number	Status	Study title
Ibogaine	NCT04003948	Recruiting	Preliminary efficacy and safety of ibogaine in the treatment of methadone detoxification
	NCT05029401	Recruiting	A study of oral ibogaine in opioid withdrawal
Ketamine	NCT00300794	Completed	Effects of ketamine on precipitated opioid withdrawal under general anesthesia
	NCT04892251	Recruiting	Ketamine-assisted psychotherapy for opioid use disorder
	NCT05051449	Recruiting	Ketamine for OUD and comorbid depression (OUDCD)
	NCT04177706	Recruiting	Ketamine for the treatment of opioid use disorder and depression
Psilocybin	NCT05242029	Not yet recruiting	Psilocybin for opioid use disorder in patients on methadone maintenance with the ongoing opioid use
	NCT05585229	Not yet recruiting	Standardized natural psilocybin-assisted psychotherapy for the tapering of opioid medication
	NCT04161066	Recruiting	Adjunctive effects of psilocybin and a formulation of buprenorphine
MDMA	NCT05219175	Not yet recruiting	MDMA for co-occurring PTSD and OUD after childbirth

## Ketamine

During the 1950s, phencyclidine (PCP) was widely synthesized and used as a dissociative anesthetic (Naughton et al., 2014). The drug was removed from the market in 1978 because of its high abuse potential along with psychodysleptic side effects (delusions, hallucinations, and out-of-body experiences). Ketamine, a derivative of PCP, gradually replaced PCP as a clinical anesthetic during the 1970s (Dundee et al., 1970). As with PCP, ketamine does induce psychodysleptic effects, but to a lesser extent, while maintaining hemodynamic stability (Zanos et al., 2018). Because of these unique side effects, ketamine is primarily used as an anesthetic in veterinary medicine, although it is also utilized as one of the preferred anesthetic agents in humans for certain specific applications, such as pediatrics, burn victims, and hemodynamically compromised patients (Morgan et al., 2012; Nowacka and Borczyk, 2019). Beyond its anesthetic uses, ketamine is also commonly used as an analgesic. Adding ketamine to opioids reduces respiratory depression and prevents hyperalgesia during acute pain management (Chawarski et al., 2020). In this way, ketamine is already being utilized to lower the dosage of prescription opioids needed in treatment, which may assist in preventing more patients from becoming dependent on opioids in the first place.

The most notable and recent application of ketamine as a therapeutic agent, however, is its utilization as an antidepressant in conjunction with psychotherapy. Ketamine has been shown to be highly effective in treating refractory major depressive disorder along with depression-resistant suicidal thoughts, with a rapid and long-lasting remission or reduction in the symptoms (Serafini et al., 2014; Reinstatler and Youssef, 2015; Loo et al., 2016). Additionally, it has become evident that ketamine shows potential to treat opioid, alcohol, and cocaine addiction (Krupitsky and Grinenko, 1997; Krupitsky et al., 2007; Dakwar et al., 2014). In an early study by Kruptisky and Grinenko, 70 detoxified heroin-addicted patients were randomized to receive high-dose ketamine (2 mg/kg intramuscularly) or a sub-psychedelic low dose of ketamine (0.2 mg/kg intramuscularly) alongside psychotherapy. The purpose of these psychotherapy sessions was to connect the ketamine experience with the patients’ interpersonal problems and carrying out a life without heroin usage. High-dose ketamine showed a significantly higher reduction in abstinence at 24 months *vs.* the low-dose group in addition to a significant reduction in cravings immediately after ketamine infusion (Krupitsky and Grinenko, 1997). A follow-up study compared the efficacy of a single dose *vs.* multiple sessions of ketamine-assisted psychotherapy for heroin addiction (Krupitsky et al., 2007). After one year, 50% percent of patients remained abstinent in the multiple-dose group *vs.* 22% in the single-dose group (*p* <0.05). These initial studies serve to indicate that ketamine shows potential to treat OUD, although higher (psychedelic) doses and multiple follow-up sessions may be necessary to maintain abstinence.

### Potential mechanisms of action for ketamine as a treatment for OUD

As a dissociative anesthetic and analgesic, ketamine works by inhibiting open NMDA receptors in a non-competitive manner, which is considered to play a role in its utility in both depression and SUDs (Franks and Lieb, 1994; Naughton et al., 2014; Strasburger et al., 2017). NMDA normally serves as a binding site for glutamate. Glutamate dysfunction is thought to be one of the pathways involved in SUDs and other neuropsychiatric disorders. Addiction research has shown that metabotropic glutamate receptor 2 (mGlu2), which is abundant in the mesocorticolimbic system, medial PFC, and the nucleus accumbens (nAC), has a reduced function in response to prolonged exposure to drugs, such as alcohol, resulting in altered reward pathways and increased drug-seeking behaviors (Domanegg et al., 2023). Utilizing compounds with NMDA receptor antagonist activity, such as ketamine or ibogaine, works to restore the potential glutamate imbalance and produce synaptic improvements, ultimately allowing one to learn new behaviors (Browne and Lucki, 2013; Strasburger et al., 2017). The rapidity and relatively long-term efficacy of ketamine for depression (and potentially SUDs) have been linked, like ibogaine/noribogaine and classic psychedelics, to a rapid synthesis of brain- and/or glial-derived growth factors along with the activation of the mTOR signaling pathway, leading to synaptogenesis (Li et al., 2010; Autry et al., 2011; Ly et al., 2018; Aleksandrova and Phillips, 2021). In addition to ketamine’s activity at NMDA receptors, recent studies have shown that its activation of µ-opioid receptors, in particular, may be essential to its initial antidepressant effects (Heifets et al., 2021). Although a recent clinical trial investigating the effects of ketamine on precipitated opioid withdrawal (NCT00300794, Table 2) may help answer whether this opioid activity assists with craving and withdrawal symptoms similar to ibogaine, this is an active area of research. Interestingly, other NMDA antagonists that have been developed in order to treat depression, similar to ketamine, without either the opioid or hallucinogenic activity, have not shown as much efficacy in either neurogenesis or long-lasting symptom reduction (Autry et al., 2011; Browne and Lucki, 2013). As with both ibogaine and classic psychedelics, ketamine pharmacology is complex, interacting with multiple neurotransmitters and receptors (Kohrs and Durieux, 1998; Udesky et al., 2005; Mion and Villevieille, 2013; Pacheco Dda et al., 2014). Additionally, similar to the other psychoplastogens discussed here, researchers are still unable to determine how much of ketamine’s efficacy in depression (and possibly SUDs) is reliant on the hallucinogenic and psychotherapeutic components of ketamine-assisted psychotherapy. In Krupitsky’s two-year follow-up study, it was noted that high-dose ketamine, while significantly improving heroin abstinence rates and decreasing cravings, also increased non-verbal emotional attitudes, as compared with a low dose (Krupitsky et al., 2002). Interestingly, both low and high doses improved patients’ symptoms of anxiety, depression, and anhedonia, while increasing their spirituality. Additionally, a study of the effects of ketamine on motivation to quit and cue-induced cravings in cocaine-dependent individuals found that ketamine significantly enhanced motivation for changing problematic behaviors (Dakwar et al., 2014). This is particularly notable as these patients were only subject to a short mindfulness exercise prior to treatment, as opposed to a full psychotherapy session. Moving forward, it is imperative that researchers seeking to develop new compounds, whether to improve safety or to eliminate the altered states of consciousness from the equation, thoroughly investigate the activity of these older medicines. If researchers can identify the pharmacological action and chemical moiety responsible for the enhanced motivation seen in ketamine, for example, it will ensure that this aspect of the potential therapeutic effect can be incorporated into the new compound or can at least be methodically studied by alternate incorporation and removal.

### Safety, guidelines, and future considerations for ketamine in the treatment of OUD

Despite the lower potency and shorter half-life when compared to its parent drug PCP, ketamine is still used recreationally as well to produce feelings of weightlessness, out-of-body experiences, and visions (Curran and Morgan, 2000). Interestingly, regardless of this fact, unlike ibogaine and classic psychedelics, ketamine is a Schedule III drug in the United States. Therefore, it has some advantages, in terms of our knowledge, of the safety profile and clinical guidelines as compared with other psychoplastogens (Sanacora et al., 2017; Cohen et al., 2018; Schwenk et al., 2018). Although it remains to be determined what the ideal dose of ketamine for OUD would be, the known side effects of doses currently used in treating depression include transient cardiovascular alterations, such as an increased heart rate and blood pressure, mild respiratory depression, and dissociative or hallucinogenic effects (Mion and Villevieille, 2013; Sanacora et al., 2017). Several clinical trials of ketamine for OUD alone and with comorbid depression are already underway (Table 2). The results of these trials will help further establish the safety and efficacy of this potential treatment, and hopefully pave the way for future research and trials with other psychoplastogens.

## Classic psychedelics

5-HT_2A_ agonists, otherwise known as “classic psychedelics,” including lysergic acid diethylamide, psilocybin, and ayahuasca, have been known for nearly three-quarters of a century to provide benefit to those suffering from mental health disorders, including addiction (Abuzzahab et al., 1971; Savage and McCabe, 1973; Krebs and Johansen, 2012; Dos Santos et al., 2016). Unfortunately, a rise in the recreational use of these compounds and the resulting social and legal fallout led to the inability of researchers to study, and subsequently patients to benefit from, these drugs for 30+ years. In the past 25 years, however, there has been a resurgence of interest in psychedelics and the potential they hold for the rising incidence of mental health conditions in the United States and throughout the world. This renewed interest is not only capturing the attention of researchers and medical or mental health providers, it is also gripping the public, with 7.4 million people nationwide aged ≥18 years using hallucinogens in 2021 (SAMHSA, 2022).

The recent public interest, contrary to that of the 1960s, is being harnessed by researchers interested in furthering the therapeutic potential of psychedelics. Utilizing data from the National Survey of Drug Use and Health (NSDUH) from 2008–2013, Pisano et al. found that psychedelic use decreased the odds of past year opioid dependence by 27% and opioid abuse by 40% (Pisano et al., 2017). This was surpassed only by cannabis use, which reduced the risk of past year opioid abuse by 55%. In a follow-up study, using NSDUH data from 2015–2019, another group found that, of classic psychedelics, only psilocybin reduced the risk of OUD by 30%, while both cannabis and MDMA increased the risk (Jones G. et al., 2022). In Canada, psychedelic use among marginalized women who used opioids was found to significantly decrease the hazards of suicidal ideation or attempt (Argento et al., 2018), while recent psychedelic use was associated with 55% reduced odds of daily opioid use among a population of people who use illicit drugs (Argento et al., 2022). These studies, combined with early reports and recent clinical trials of classic psychedelics for the treatment of depression (Becker et al., 2022; Goodwin et al., 2023), anxiety (Uthaug et al., 2021; Holze et al., 2023), PTSD (Mitchell et al., 2021), and addiction (Abuzzahab et al., 1971; Dos Santos et al., 2016; Johnson et al., 2017; O'Shaughnessy et al., 2021; Sessa et al., 2021; Bogenschutz et al., 2022; Nicholas et al., 2022), all indicate that this class of drugs shows potential to be a novel alternative therapy for OUD.

### Potential mechanisms of action for psychedelics as a treatment for OUD

As with ibogaine, LSD, psilocybin, and ayahuasca each exhibit their own unique pharmacological profile, interacting with a wide variety of neurotransmitters and receptors within the brain. They are typically classed together, however, as they are all 5-HT_2A_ receptor agonists, and as such, they produce an altered state of consciousness together with rapid neuroplasticity. As potent psychoplastogens, LSD, psilocin (the active metabolite of psilocybin), and *N-N*-dimethyltryptamine (DMT; the main psychoactive component of ayahuasca) have all been shown to increase structural and functional neuritogenesis, spinogenesis, and synaptogenesis within cortical neurons (Ly et al., 2018; Shao et al., 2021; Olson, 2022). This effect, purportedly driven by intracellular 5-HT_2A_ receptors and mTOR and TrkB signaling pathways, appears to be both rapid and persisting (Shao et al., 2021; Vargas et al., 2023). This psychoplastogen effect could account for the reported long-lasting efficacy of these compounds, as noted after only one to three doses. Additionally, as with ketamine, psychedelics may restore glutamate transmission in the PFC. In the case of 5-HT_2A_ agonists, it has been found that 5-HT_2A_ and mGlu2 are capable of regulating each other’s downstream signaling pathways via monovalent crosstalk or heteromer formation (Duman et al., 2021). One theory for neuroplastic-induced efficacy with regards to SUDs is a top–down approach, wherein changes to the PFC via neurogenesis, increasing mGlu2 and decreasing 5-HT_2A_, leads to increased inhibitory control and decreased stress reactivity within dorsal raphe nuclei and the extended amygdala with a subsequent decreased stress response and stress-induced neurodegeneration at the hypothalamus–pituitary–adrenal axis (Urban et al., 2023). The neuroplastic changes in the PFC may additionally signal to the ventral tegmental area and the nAC to decrease corticoaccumbal transmission, thereby reducing impulsivity and drug-seeking behaviors.

Nevertheless, as it is with ibogaine, it is currently not possible to separate the known pharmacological effects from the psychological effects caused by psychedelics, producing altered states of consciousness. Although each of the psychedelics produce their own unique “trips” in terms of duration, physical side effects, and hallucinatory manifestations, the impact of the experience on the user tends to be profound and, for the most part, highly beneficial. Higher psychedelic doses, mystical-type experiences, and greater personal insights are all correlated with a more favorable outcome (Argento et al., 2019; Garcia-Romeu et al., 2019; Hamill et al., 2019). Indeed, even when controlling the intensity of the drug’s effects, mystical-type subjective effects have been found in some studies to be “necessary” for the enduring beneficial efficacy of the drug (Garcia-Romeu et al., 2014; Yaden and Griffiths, 2021). In the psilocybin tobacco cessation study, the authors likened the effect of the mystical experience to an “inverse PTSD-like experience,” in which a singular event was capable of causing lasting behavioral and likely biological benefits to the patients, with one patient stating that it left them “feeling complete as a person and physically part of all things” (Garcia-Romeu et al., 2014). It should be noted that the studies investigating these drugs for changes in mental health or addiction nearly always involve a strong psychotherapeutic component, whether in the form of a religious ceremony or Western psychotherapy. Indeed, ritual users, such as those who partake of ayahuasca as a sacrament in the Uniao do Vegetal (UDV) church, acknowledge that, for them, the two cannot be separated (Grob et al., 1996). Ayahuasca served as a catalyst for change in their lives, but the ritual and community helped solidify their resolutions and outlook on life. Similarly, post-treatment psychotherapy employed by modern researchers serves to help patients integrate the visionary experience into their lives moving forward.

### Safety, guidelines, and future considerations for classic psychedelics in the treatment of OUD

Despite multiple recent clinical trials demonstrating the efficacy of classic psychedelics for a number of neuropsychiatric conditions, these compounds remain as Schedule I drugs. Therefore, there are no set clinical guidelines with regards to patient exclusion criteria, dosing, administration, or medical monitoring, although individual states are beginning to establish these details (OHA, 2023). Likewise, the FDA recently released a draft of the guidelines for the industry to address the necessary and complicated components of conducting clinical investigations with psychedelic drugs (FDA, 2023). Nevertheless, 5-HT_2A_ agonists are generally considered safe and non-addictive. DMT has been shown to increase the heart rate, blood pressure, and anxiety, but these adverse events are mild and self-limited (D'Souza et al., 2022). Ayahuasca, which contains monoamine oxidase inhibitors, harmine and harmaline, in addition to DMT, causes similar short-term cardiovascular responses as DMT alone, along with typically inducing nausea and vomiting shortly after ingestion (Hamill et al., 2019). The emesis portion of the ayahuasca experience is often portrayed as a “cleansing” to prepare the participant for the visions to come. Adverse events reported with psilocybin include headache, nausea, dizziness, and a transient increase in BP and HR, but, as with ayahuasca and DMT, these are self-limited (Goodwin et al., 2022). A recent clinical trial of LSD for anxiety reported mild and transient adverse events during treatment, including anxiety, nausea, and headache (Holze et al., 2023). Overall, the adverse effects of psychedelics tend to be mild and transitory, while the positive benefits to neuropsychiatric disorders and a general outlook on life are rapid and long-lasting. To date, despite the potential, there have been no published results of clinical trials on classic psychedelics for the treatment of OUD, although three trials involving psilocybin and one of MDMA are in the initial and recruiting phases (Table 2). The results of these trials will hopefully add to the growing evidence of the safety and efficacy of classic psychedelics for the treatment of not only neuropsychiatric disorders and substance abuse but also of the person as a whole. Indeed, a couple of these trials (NCT05242029 and NCT05585229) are notable in the sense that they are investigating the potential of psilocybin in patients who are currently using OATs for OUD. Although many patients who seek out treatments like ibogaine, ketamine, or psychedelics for OUD do so because they have found OATs to either be ineffectual or too difficult to adhere to, it is possible that the combination of therapy may be synergistic pharmacologically or that psychoplastogens may enhance the motivation of patients to adhere to the regimen of OATs. On the other hand, the use of these compounds may assist patients in transitioning off of OATs. This is an interesting concept requiring further study.

## Conclusion

The opioid epidemic is a crisis at the national level that the government and public health authorities are attempting to combat by increasing funding and access to existing evidence-based prevention and treatment programs while alongside addressing socioeconomic and mental health factors. For patients with OUD, it is a personal battle—one that encompasses their physical and mental health, their finances, their relationships, and their whole lives. New treatment options are desperately needed that can address not only the physical addiction but also patients’ mental health and overall outlook on life. Psychoplastogens, like ibogaine, ketamine, and classic psychedelics, present a novel approach with the potential to treat the patient as a whole with rapid, long-lasting efficacy. As we continue to reevaluate these compounds as medicines rather than drugs of abuse themselves, future clinical trials are needed to establish best-practice guidelines along with their long-term efficacy and safety. Nevertheless, for those suffering with OUD, as well as their friends and family, the potential of these therapies provides hope for a better future.
